# Development of bioelectrocatalytic activity stimulates mixed-culture reduction of glycerol in a bioelectrochemical system

**DOI:** 10.1111/1751-7915.12240

**Published:** 2015-03-26

**Authors:** Mi Zhou, Stefano Freguia, Paul G Dennis, Jürg Keller, Korneel Rabaey

**Affiliations:** 1 Advanced Water Management Centre, The University of QueenslandBrisbane; 2 Key Laboratory of Industrial Ecology and Environmental Engineering (MOE), School of Environmental Science and Technology, Dalian University of TechnologyDalian, 116024, China; 3 Centre for Microbial Electrosynthesis, The University of QueenslandBrisbane; 4 Australian Centre for Ecogenomics, The School of Chemistry and Molecular Biosciences, The University of QueenslandBrisbane, QLD, 4072, Australia; 5 School of Agriculture and Food Sciences, The University of QueenslandBrisbane, QLD, 4072, Australia; 6 Laboratory of Microbial Ecology and Technology, Ghent UniversityCoupure Links 653, Ghent, 9000, Belgium

## Abstract

In a microbial bioelectrochemical system (BES), organic substrate such as glycerol can be reductively converted to 1,3-propanediol (1,3-PDO) by a mixed population biofilm growing on the cathode. Here, we show that 1,3-PDO yields positively correlated to the electrons supplied, increasing from 0.27 ± 0.13 to 0.57 ± 0.09 mol PDO mol^−1^ glycerol when the cathodic current switched from 1 A m^−2^ to 10 A m^−2^. Electrochemical measurements with linear sweep voltammetry (LSV) demonstrated that the biofilm was bioelectrocatalytically active and that the cathodic current was greatly enhanced only in the presence of both biofilm and glycerol, with an onset potential of −0.46 V. This indicates that glycerol or its degradation products effectively served as cathodic electron acceptor. During long-term operation (> 150 days), however, the yield decreased gradually to 0.13 ± 0.02 mol PDO mol^−1^ glycerol, and the current–product correlation disappeared. The onset potentials for cathodic current decreased to −0.58 V in the LSV tests at this stage, irrespective of the presence or absence of glycerol, with electrons from the cathode almost exclusively used for hydrogen evolution (accounted for 99.9% and 89.5% of the electrons transferred at glycerol and glycerol-free conditions respectively). Community analysis evidenced a decreasing relative abundance of *Citrobacter* in the biofilm, indicating a community succession leading to cathode independent processes relative to the glycerol. It is thus shown here that in processes where substrate conversion can occur independently of the electrode, electroactive microorganisms can be outcompeted and effectively disconnected from the substrate.

## Introduction

Microbial bioelectrochemical systems (BESs) can use microorganisms as the catalyst to overcome high overpotential and low specificity of electrode reactions (Rabaey and Rozendal, 2010; Logan and Rabaey, 2012). Upon developing bioelectrocatalytic activity in biocathodes, the performance of reactors can be greatly optimized in terms of energy production (Xia *et al*., 2013), hydrogen evolution (Rozendal *et al*., 2008), CO_2_ fixation to CH_4_ (Cheng *et al*., 2009) or acetate (Nevin *et al*., 2010; Zhang *et al*., 2013) in bioelectrosynthesis. Previously, we demonstrated that the conversion of glycerol to 1,3-propanediol (1,3-PDO), which is one of the oldest known biological processes (Saxena *et al*., 2009), can be stimulated by imposing a cathodic current to a mixed bacterial consortium fermenting glycerol (Zhou *et al*., 2013). This may derive from the interactions between solid electrode and cathodic bacteria; however, direct evidence of bioelectrocatalytic role in the glycerol-fed bioelectrochemical reactors was not provided thus far. In an earlier study, our group established that 1,3-PDO formation was positively correlated with *Citrobacter* species in BESs; however, reduction in yield during 9 weeks of operation (Dennis *et al*., 2013a) related to lower product yields and increased valerate production. This is opposite to the improved performance observed in a mixed-culture autotrophic biocathode after operating over 150 days (Marshall *et al*., 2013).

It is recognized that voltammetry can be a powerful tool for the investigation of bioelectrocatalytic activity in BESs reactors. Faradaic current can emerge at an early onset potential, indicating that microbial cells facilitate the electron transfer of redox reactions (Harnisch and Freguia, 2012). Voltammetric methods can thus be applied in conjunction with chemical analysis and community characterization to understand bacterial activity over time in glycerol-fed BESs. To study this, we operated and monitored a glycerol-fed BES for more than 150 days using linear sweep voltammetry (LSV) at different operational stages. Compositions of the biofilm and planktonic community were also analysed. The current study describes for the first time that bioelectrocatalytic activities relates to the cathodic electron sink shift over time in BES reactors.

## Results and discussion

### Performance of 1,3-PDO production reactors

**Fig 1:**
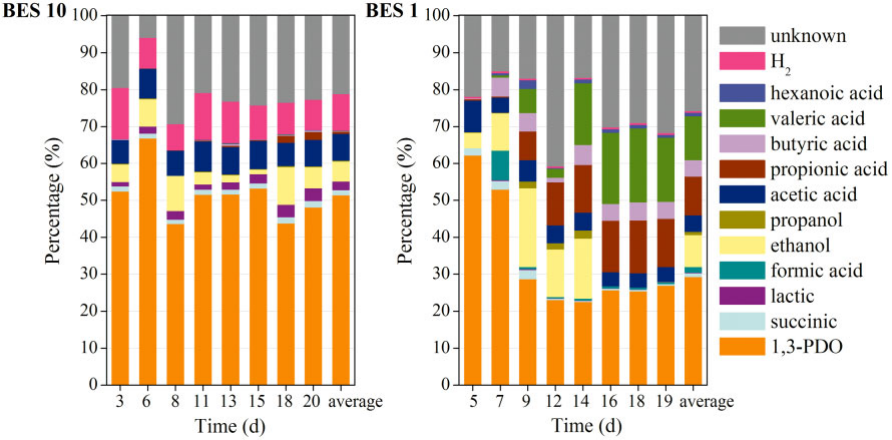
Metabolite and electron balances of two identical glycerol-fed BES reactors operated with different cathodic currents. BES 10 was started with a controlled current of 10 A m^−2^, and the current for BES 1 was 1 A m^−2^. Biomass formation was not evaluated through time as this would require removal of biofilm from the cathode.

1,3-PDO production started immediately after the inoculation and glycerol feed. To investigate the effect of different levels of electron inputs to 1,3-PDO production, two identical BES reactors were operated with different cathodic currents from the beginning of the operation. With current and glycerol as the two electron inputs, electron balances among the metabolites from both reactors from day 1 to 20 are illustrated in Fig. 1.

Glycerol contributed to 80.0 ± 4.9% of the electron inputs for BES 10 (with 10 A m^−2^ cathodic current), and eventually 51.5 ± 7.0% of the electron inputs went to 1,3-PDO production. In BES 1, when the cathodic current was 1 A m^−2^, only 1.5 ± 0.2% of the electrons were from current and 29.4 ± 10.7% of the electron inputs were stored in 1,3-PDO. These corresponded to a yield of 0.57 ± 0.09 mol PDO mol^–1^ glycerol with −10 A m^−2^ and 0.27 ± 0.13 mol PDO mol^−1^ glycerol at −1 A m^−2^. Given that a higher cathodic current represents more electron input, this demonstrated that electrons pushed glycerol metabolism towards 1,3-PDO production. It is interesting to notice that for the first 7 days of BES 1, 1,3-PDO production was comparable with that of BES 10 (accounted for 57.7 ± 6.5% of the electron inputs). However, the yield decreased afterwards, and propionic acid production emerged together with butyric and valeric acid formation. The propionate formation is an electron neutral process at cellular level (Stadtman *et al*., 1949; Zeng, 1996; Barbirato *et al*., 1997), implying that flux towards glycerol reduction was triggered and maintained by a higher reductive current at this stage. The unaccounted electrons should be mainly used for biomass formation, which can take up to 20% electron input (Zhou *et al*., 2013).

**Fig 2:**
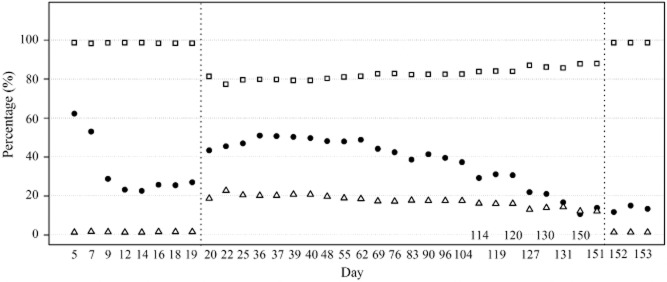
Electron inputs and 1,3-PDO production of the cathodic fermentation in BES 1 with continuous flow operation at different cathodic currents. The reactor started with a cathodic current of 1 A m^−2^, and glycerol accounted for 98.5 ± 0.2% of the electron inputs. To test if 1,3-PDO yield is positively correlated to the current input, the cathodic current applied was enhanced to 10 A m^−2^, and 17.5 ± 2.9% of the electrons derived from current from day 20 until day 151. 1,3-PDO production decreased gradually, and after day 151, when the cathodic current was switched back to 1 A m^−2^, no significantly difference regarding 1,3-PDO production was observed. The last three points of the figure indicate the average value of day 152 and day 153.

To substantiate this observation, the cathodic current for BES 1 was increased to 10 A m^−2^ after day 19. 1,3-PDO production increased immediately to 44.0 ± 1.0% of the total electron inputs (Fig. 2, from day 20 to day 22). This provided further evidence that the reductive conversion of glycerol was directly or indirectly stimulated by the current applied, which is subject to manual manipulation. It is noteworthy that with a cathodic current of 10 A·m^–2^, the cathodic potential can be as low as −1.4 V, which enables hydrogen evolution at carbon electrodes. The cathodic potential was −0.80 ± 0.03 V with −1 A m^–2^ current. Indeed, more hydrogen was captured in the headspace of BES 10 compared with BES 1 (Fig. 1). Providing hydrogen to fermentative bacteria can promote 1,3-PDO production to some extent according to our previous study (Zhou *et al*., 2013), hence the need to understand the nature of the bioelectrocatalytic activity.

### Bioelectrocatalytic activity developed in the biofilm

**Fig 3:**
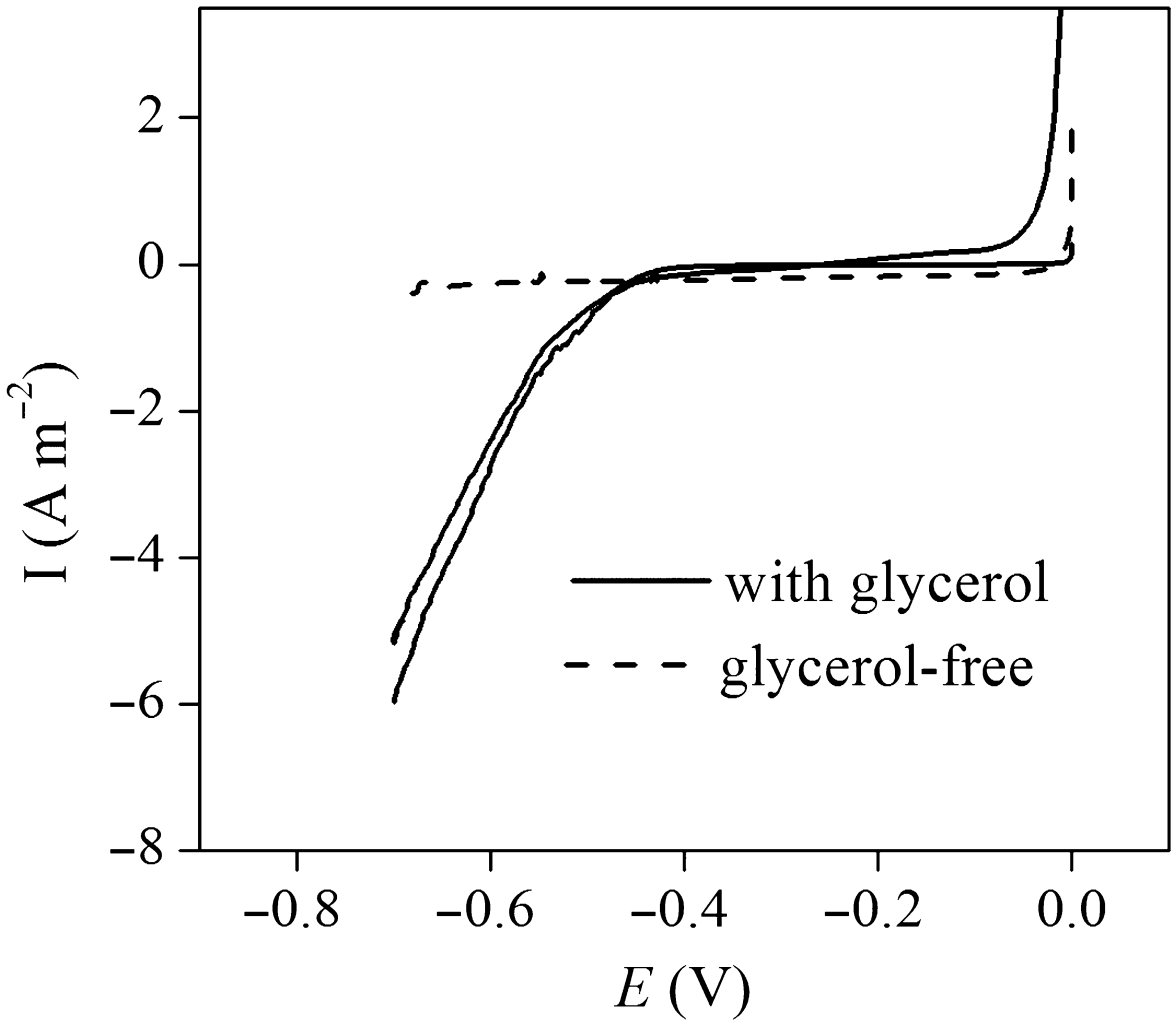
Voltammogram of the cathodic biofilm from day 89 to day 93. The solid lines: biological cathode with glycerol (here we show the results from two independent scans conducted at day 89 and day 93 respectively); dash line: biological cathode without glycerol, which was performed at day 91.

The current of BES 1 was kept at −10 A m^−2^ after day 19. From day 20 to day 83, 46.7 ± 3.7% of the electron inputs went to 1,3-PDO (Fig. 2). To support our hypothesis that 1,3-PDO production by the mixed-culture biofilm was sustained by electrons released at the electrode, LSV tests were conducted from day 89 to day 93. To prevent electrochemical hydrogen evolution during the LSV scans, the chosen potential window was 0 V to –0.7 V, because the onset potential for hydrogen evolution with an identical graphite electrode was more electronegative than –0.7 V (Fig. S2). Moreover, a changing potential enables two types of current, i.e., capacitive current and Faradaic current. To avoid the interference from capacitive current, which is not related to redox reactions and may mask the Faradaic current (Harnisch and Freguia, 2012), a scan rate as low as 0.1 mV s^−1^ was adopted for all the voltammetric scans in this study. According to the voltammetric curves (Fig. 3), the capacitive response was low due to this approach.

Figure 3 illustrates the interesting observation that when glycerol was depleted, Faradaic effect was not observed within the scan range, and the current density was near 0 A m^−2^. In contrast, a reductive current with an onset potential of –0.46 V was observed in the presence of glycerol, and current increased rapidly with more negative polarization. Cathodic current should only emerge when reductive actions take place, with the presence of an electron sink. This suggests that glycerol addition leads to the availability of electron accepting compounds, which could be glycerol or one of its derivatives. One could argue that the cathodic reaction was water electrolysis as the onset potential for hydrogen evolution can shift positively when the cathodic pH decreases due to the formation of carboxylic acid from glycerol; however, hydrogen was not detected in the gas bags connected to the headspace of the reactor during these LSVs. In addition, to reach the observed shift, the cathode pH should have decreased by five units according to the Nernst equation, which is highly unlikely given that the cathode bulk fluid was controlled at pH 5.5 and the activity of the biofilm remained unaffected after the LSV tests (Fig. 2, day 90). Therefore, by correlating reductive current to the presence or absence of glycerol, we demonstrated that it is very likely that glycerol or the fermentative products were the cathodic electron sink at this stage of the biofilm. Moreover, the abiotic voltammogram exhibited no current response at −0.46 V with glycerol (Fig. S2), excluding the possibility of glycerol receiving electrons directly from the cathode. These results further imply that with glycerol or the fermentative products as the electron acceptor, the cathodic biofilm facilitated the reductive reaction, and bioelectrocatalytic activity was developed in the glycerol-fed BESs. A similar observation was reported in a methanogenesis biocathode with direct electron transfer previously (Cheng *et al*., 2009).

### Long-term performance of reactors and shift of bioelectrocatalytic activity

**Fig 4:**
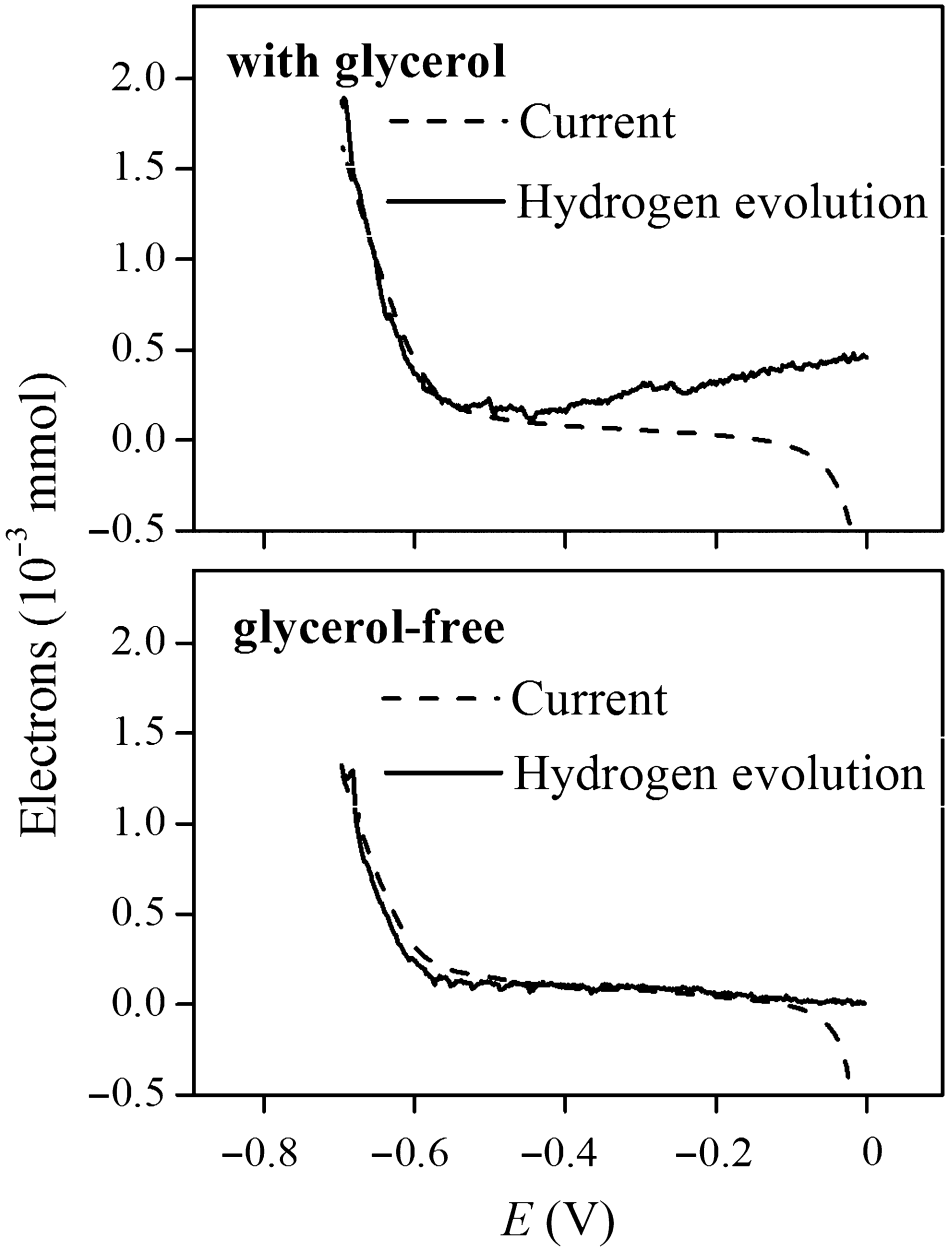
Electrons supplied and used for hydrogen formation during linear sweep voltammetry tests in a late stage of the experiments (day 164–166). The dashed lines in the two figures show the electron flow as cathodic currents, and the solid lines illustrate electrons occupied by hydrogen production under glycerol and glycerol-free conditions.

To investigate the long-term performance of glycerol-fed BES reactors, the cathodic current of BES 1 was maintained at 10 A m^−2^ and continuously operated for more than 150 days. 1,3-PDO production went down gradually with time (Fig. 2). At day 150, 1,3-PDO only accounted for 12.3 ± 2.2% of the supplied electrons with –10 A m^−2^ current, and 13.4  ±  2.4% electrons ended up in 1,3-PDO with –1 A m^−2^ current indicated by duplicate test (Fig. 2, day 152 and 153). The effect of cathodic current was marginal after long-time operation. The cathodic products profile should be related to the electrochemical activity of bacteria. To investigate this, LSVs were recorded again at day 164 and 166 under exactly the same procedure as previously. Contrarily to the first experiments, considerable amounts of hydrogen gas were produced and the onset potentials for cathodic current were identical irrespective of the presence of glycerol. We calculated and compared the electron input as current and used for hydrogen evolution during the LSVs (Fig. 4).

As can be seen in Fig. 4, electrons were almost exclusively used for hydrogen evolution at both conditions (accounting for 99.9% and 89.5% of the electrons transferred at glycerol and glycerol-free conditions respectively). The presence of glycerol did not impact the onset potentials, which were now lower, i.e., −0.58 V for both conditions. Apparently, hydrogen became the dominant electron sink. Although we cannot completely rule out the possibility that a minor amount of hydrogen was produced in the previous LSV tests from day 89, given the different onset potentials of the LSV tests at different operational stage, it is highly likely that at this stage of operation, bioelectrocatalytic activity shifted to hydrogen evolution. Other gaseous products, i.e., CO_2_ and CH_4_ were also monitored, their amounts were negligible in the test without glycerol and exhibited no obvious links between current in the test with glycerol (Fig. S3). Previously, it has been proven that the 1,3-PDO yield from supplying hydrogen directly to fermentative bacteria was significantly lower comparing with the level with current (Zhou *et al*., 2013). Therefore, two LSV tests performed at different operational stages suggest the development and shift of bioelectrocatalytic activity, which should relate to the changed reactor performance.

### Bacterial community and selectivity posed by the cathodic electrode

The performance of BES 10 was stable during the first 30 days. To investigate the bacterial populations, BES 10 was terminated at day 30, and the biofilm and planktonic population were analysed. Populations with relative abundance of more than 1% of the communities are illustrated in Fig. S4. Similar as the previous study with glycerol as the cathodic feed (Dennis *et al*., 2013a), the biofilm was dominated by an operational taxonomic unit that was closely related to *Citrobacter* spp., which represented 80.3% of the community. However, the planktonic community exhibited a distinct composition, with *Citrobacter* representing only 29.4% relative abundance and several other dominant operational taxonomic units. Bacterial populations could be correlated to the products of reactors, but their bioelectrocatalytic role is still unknown. Although transferring electrons to a solid electrode was reported in *Citrobacter* species (Xu and Liu, 2011), isolating microorganisms from the glycerol-fed biocathode would be necessary to unequivocally relate *Citrobacter* and bioelectrocatalytic activity, which is outside the scope of the present study.

At day 159, fluorescent in-situ hybridization (FISH) was used as the technique to evaluate whether similar populations were still present in the reactors. The FISH showed a dramatic decrease of gammaproteobacterial (*Citrobacter*) populations in the cathodic biofilm and the presence of *Methanosaeta* (methanogens) in the planktonic population (Fig. S5). This is consistent with the finding that methane was detected since day 65 and exhibited an increasing trend (data not shown). Although FISH is not strictly quantitative, it establishes the relationship between *Citrobacter* and 1,3-PDO production in BES reactors, as well as the dynamics of cathodic population in glycerol-fed BES reactors.

With continuous supply of cathodic current over 150 days, glycerol reduction decreased and could not be recovered and bioelectrocatalytic activity shifted over time. This was different from reports on the biocathodes capturing CO_2_ to produce methane (Van Eerten-Jansen *et al*., 2012) or acetate (Marshall *et al*., 2013), where stable and even improved performances were observed over long periods. This likely relates to the strict dependency of the latter mentioned processes on the cathode, whereas fermentative processes can occur irrespective of the cathode. In addition, the presence of multiple side products, enabling growth of different bacteria can be implicated. Bioelectrocatalytic glycerol reduction and hydrogen evolution are thus two coexisting electron sinks. Following our results, it appears that a fermenting population established on top of the electroactive biofilm, limiting the accessibility of glycerol to the biofilm, and thus forcing a redirection of cathode-associated processes towards hydrogen evolution. This highlights the need for either pure cultures to catalyze the cathode reaction, or an inhibition of growth of the bacteria without leading to ATP accumulation which will be challenging at best.

## Experimental procedures

### Reactors and operation

Two identical BESs were constructed as previously described (Zhou *et al*., 2013). The electrodes were graphite plates (5 × 20 cm, Morgan AM&T, UK), and the anode and cathode compartment were separated by a cation exchange membrane (surface area: 100 cm^2^, Ultrex CMI-7000, Membrane International, USA). The cathodes were inoculated with a microbial community obtained from a sewage sludge fermenter (Dennis *et al*., 2013a). During the continuous mode operation, the anode compartments were continuously supplied with a phosphate buffer (6 g l^−1^ Na_2_HPO_4_, 3 g l^−1^ KH_2_PO_4_, pH 7.1), and the biocathodes were fed with modified M9 medium (Rabaey *et al*., 2005) supplemented with 64 mM glycerol. A hydraulic retention time of 8.1 h and recirculation rate of 100 ml min^−1^ were adopted for both chambers. Electrochemical parameters were controlled and monitored by a VMP-3 potentiostat (Bio-Logic SAS, France) with Ag/AgCl reference electrodes [assumed +0.197 V versus standard hydrogen electrode (SHE)] inserted in the cathode compartments. Potentials are presented relative to the SHE throughout this manuscript. The two identical reactors were started with a controlled current of –10 A m^−2^ (BES 10) or –1 A m^−2^ (BES 1). At day 19, the current for BES 1 was also switched to –10 A m^−2^. At day 30, BES 10 was shut down for the characterization of biofilm and planktonic microbial communities.

### Metabolite analysis

The metabolites in the cathode liquid phase were analysed after sampling and filtration with 0.22 μm sterile filters. High performance liquid chromatography was used to determine glycerol and 1,3-PDO concentrations (Dennis *et al*., 2013a), whereas concentrations of volatile fatty acids and alcohols were measured with gas chromatography (Rabaey *et al*., 2010). Gaseous products were collected by gas bags and the compositions, i.e. H_2_, CH_4_ and CO_2_, were analysed by a gas chromatography equipped with a thermal conductivity detector as described previously (Tait *et al*., 2009). The metabolite data were further used to calculate electron balances in bioelectrochemical reactors, with current and glycerol as the electron inputs. The detailed calculation is presented in the Supporting Information.

### Voltammetry tests

LSV test was performed with BES 1 at day 89 and day 93 with 25 mM glycerol, which was the average concentration in the effluent of biocathode during the continuous operation. LSV was also performed at day 91 in glycerol-free condition, which was achieved by pre-feeding the cathode with fresh M9 glycerol-free medium for three hydraulic retention times. At day 164 and day 166, LSVs were conducted again with the biocathode of BES 1 connected to a titration and off-gas analysis (TOGA) sensor for online gas measurements. TOGA has been used for the investigation of electron fluxes in bioelectrochemical reactors (Freguia *et al*., 2007; Virdis *et al*., 2009). In all LSV tests, the biocathode and anode acted as the working and counter electrodes respectively. A potential window of 0 V to −0.7 V and a scan rate of 0.1 mV s^−1^ were adopted. Mass-transfer effects were minimized by recirculation during the tests.

### Microbial community characterization

At day 30, the biofilm of BES 10 was transferred to bead-beating tubes using a sterile glass microscope slide. Moreover, planktonic microorganisms from 50 ml of the cathodic electrolyte were recovered by centrifugation at 4000 r.p.m., 25 min. Cell pellets were then re-suspended in 0.5 ml of molecular biology grade water and transferred to bead-beating tubes for nucleic acid extraction. All samples were immediately frozen by immersion in liquid nitrogen and then stored at −80°C. DNA was extracted using a MO-BIO PowerBiofilm DNA Isolation Kit and then universal bacterial and archaeal 16S rRNA gens were amplified by polymerase chain reaction (PCR) using the primers 926F (5′-AAACTYAAAKGAATTGACGG-3′) and 1392R (5′-ACGGGCGGTGTGTRC-3′) as previously described (Cayford *et al*., 2012; Dennis *et al*., 2013b). Amplicons were purified using a QIAquick PCR purification kit (Qiagen), quantified using a Qubit fluorometer with a Quant-iT dsDNA BR Assay Kit, normalized to 25 ng μl^−1^ and then pooled for 454 pyrosequencing. Sequencing was performed at the Australian Centre for Ecogenomics, The University of Queensland. Sequences were quality filtered, denoised, clustered (97%) and assigned GreenGenes taxonomy as previously described (Carvalhais *et al*., 2013; Dennis *et al*., 2013b). The resulting tables with the abundance of different operational taxonomic units and their taxonomic assignments in each sample were then normalized to 800 sequences per sample for comparisons of diversity.

At day 159, FISH analysis was conducted with the cathodic biofilm and planktonic. Slides were hybridized with EUB Mix (for Bacteria), GAM Mix (for Gammaproteobacterial), ARC 915 (for Archaea) and MX 825 (for *Methanoseata*). Fluorescent DNA probes were visualized with a confocal laser-scanning microscope, and images were analysed using daime version 1.2 as described previously (Barr *et al*., 2010).

## Supporting Information

**Text S1.** Calculations of electron balance for BES reactors with continuous glycerol and current inputs. **Fig. S1.** Glycerol reduction and 1,3-PDO production in BES 10 until day 30. **Fig. S2.** Abiotic linear sweep voltammetry test. **Fig. S3.** Gaseous products during LSV scan from day 164 to day 166. **Fig. S4.** Microbial composition of the biofilm and planktonic community. **Fig. S5.** FISH image for the cathodic biofilm at day 159. **Table S1.** Cathodic potentials of both BES 10 and BES 1 under galvanostatic operation.
