# Supplementary material for: Development of bioelectrocatalytic activity stimulates mixed-culture reduction of glycerol in a bioelectrochemical system
Source: Microb Biotechnol. 2015 Mar 26;8(3):483–9. doi: 10.1111/1751-7915.12240 (PMC4408180; doi:10.1111/1751-7915.12240)
Supplement: Supplementary file 1 [file mbt20008-0483-sd1.docx]

***Supporting Information***

**Development of bioelectrocatalytic activity stimulates mixed-culture reduction of glycerol in a bioelectrochemical system**

Mi Zhou*^a,b^*, Stefano Freguia*^a,c^*, Paul G. Dennis*^a,d,e^*, Jürg Keller*^a^*, Korneel Rabaey *^f, a,c^**

*^a^Advanced Water Management Centre, The University of Queensland, Brisbane, QLD 4072, Australia;*

*^b^Key Laboratory of Industrial Ecology and Environmental Engineering (MOE), School of Environmental Science and Technology, Dalian University of Technology, Dalian 116024, China;*

*^C^Centre for Microbial Electrosynthesis, The University of Queensland, Brisbane, QLD 4072, Australia;*

*^d^Australian Centre for Ecogenomics, The School of Chemistry and Molecular Biosciences, The University of Queensland, Brisbane, QLD 4072, Australia;*

*^e^School of Agriculture and Food Sciences, The University of Queensland, Brisbane, QLD 4072, Australia;*

*^f^Laboratory of Microbial Ecology and Technology, Ghent University, Coupure Links 653, 9000 Ghent, Belgium.*

Number of pages: 8

Number of figures: 5

Number of tables: 1

**Text S1. Calculations of electron balance for BES reactors with continuous glycerol and current inputs**

Electron balances were calculated as below:

$$14\times{\Delta M}_{\mathrm{glycerol}}+\frac{\int It}{F}=\sum k_{i}\times{\Delta M}_{i, l}+\sum n_{i}\times{\Delta M}_{i, g}$$

In the equations,${\Delta M}_{i, l}$ and ${\Delta M}_{i, g}$ are the production of metabolites in the liquid phase and gas phase over a batch (mmol), respectively, $k_{i}$ and $n_{i}$ are the respective number of electrons per mole of products in the liquid phase and the gas phase (14 is the value for glycerol). *I* is the current through the BES (C/s), *t* is the time and F is the Faraday constant (96,485 C/mol).

1,3-propanediol took up 51.5 ± 7.0% of the electrons in BES 10, and 29.4 ± 10.7% of the electron fed flowed to 1,3-PDO in BES 1. The by-products were ethanol and volatile fatty acids. An average of 21.09 ± 3.09% and 25.72 ± 9.70% of the electrons were unaccounted for BES 10 and BES 1 respectively, which may end up in biomass formation. According to our previous study,[^1^](#_ENREF_1) in which we investigated the whole electron flux in a similar system equipped with a titration and off-gas analysis sensor, biomass formation accounted ~20% of the total electron inputs during the steady-state operation. It should be even higher during the acclimation period, when biofilm formation occurs.

**Fig. S1. Glycerol reduction and 1,3-PDO production in BES 10 until day 30**

**
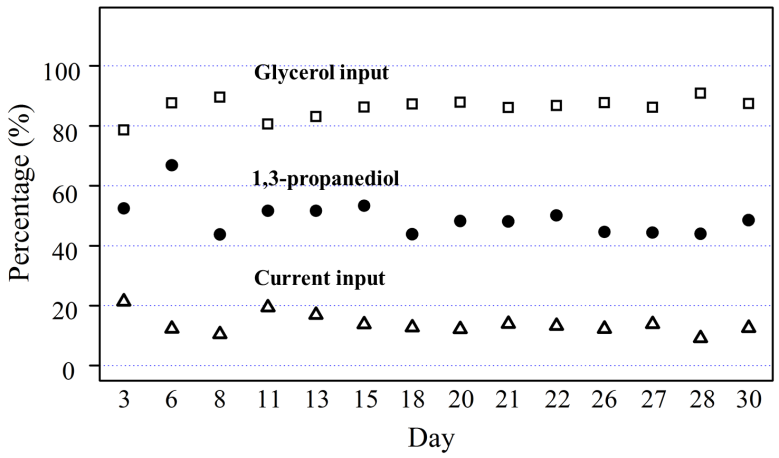
**

Figure S2. Electron inputs and 1,3-PDO production of the cathodic fermentation in BES 10 with continuous flow operation. The cathodic current was 10 A m^-2^ throughout the tests. At day 30, BES 10 was terminated for population analysis.

**Table S1. Cathodic potentials of both BES 10 and BES 1 under galvanostatic operation**

| Reactors | Operation period | Current (A m^-2^) | Cathodic potential (V vs SHE) |
| --- | --- | --- | --- |
| BES 10 | Day 1-30 | -10 | ‑1.37 ± 0.04 |
| BES 1 | Day 1-19 | -1 | -0.80 ± 0.03 |
| BES 1 | Day 19-22 | -10 | -1.438 ± 0.02 |
| BES 1 | Day 150-151 | -10 | -1.19 ± 0.02 |
| BES 1 | Day 152-153 | -1 | -0.60 ± 0.03 |

**Fig. S2. Abiotic linear sweep voltammetry test**


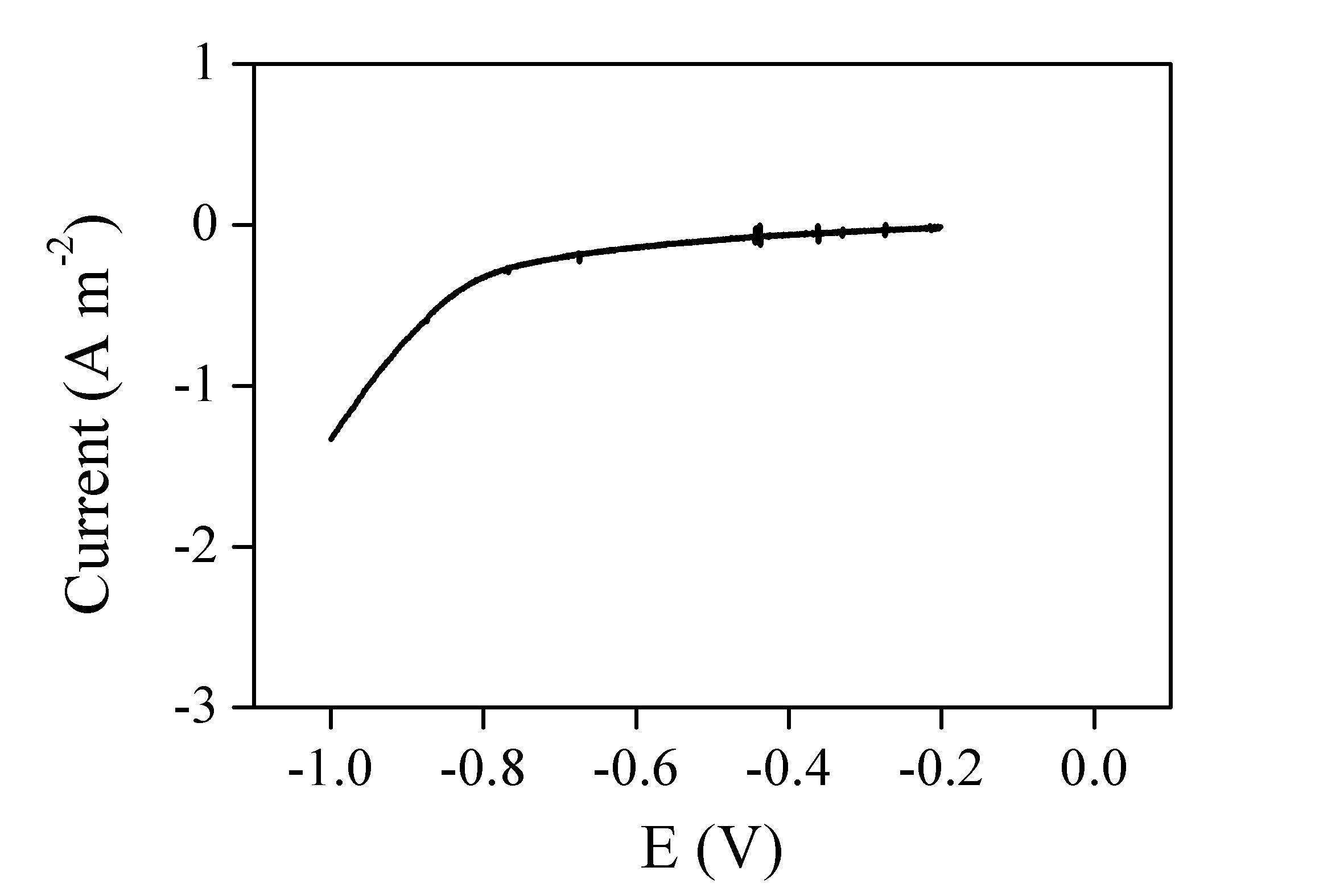


Fig. S2. Linear sweep voltammetry test with abiotic cathode in the presence of glycerol. The main purpose of this test was to identify the potential for electrochemical hydrogen evolution, and this voltammogram shows that hydrogen evolution potential on an identical graphite electrode was more negative than -0.7 V (vs SHE), therefore, the chosen potential window for LSV with the tested BES reactor were 0 V to -0.7 V.

**Fig. S3. Gaseous products during LSV scan from day 164 to day 166**

**
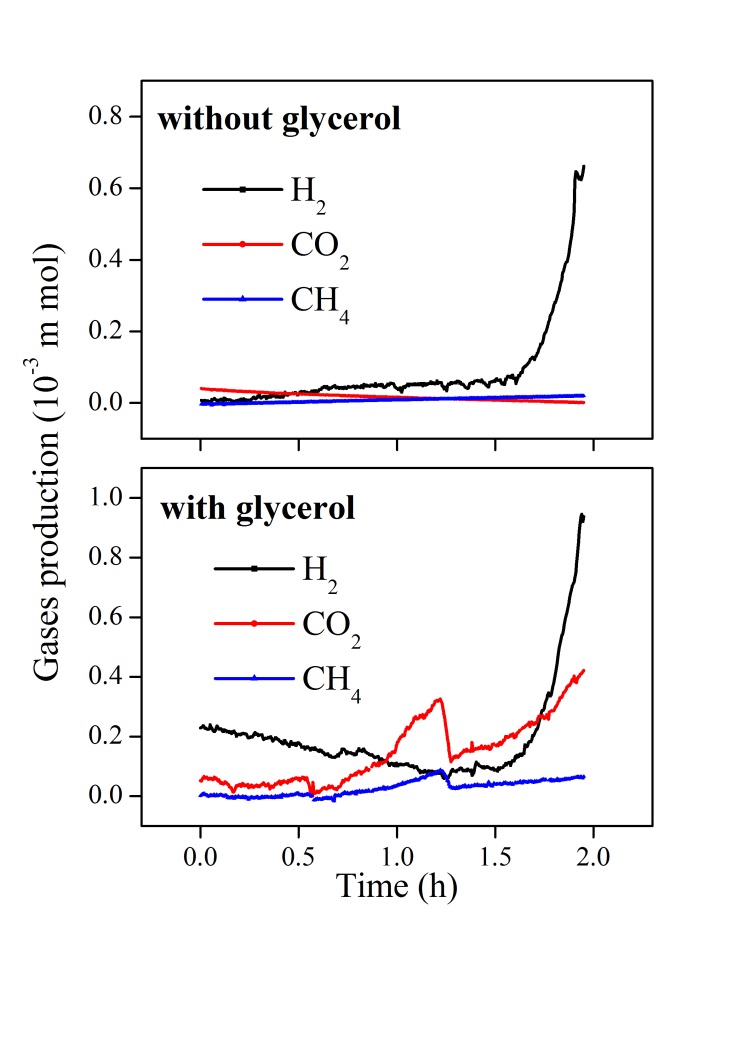
**

Fig. S3. Gaseous products during LSV scan from day 164 to 166. The test with glycerol was conducted at day 164 and the one without glycerol was performed at day 166.

**Fig. S4. Microbial composition of the biofilm and planktonic community**


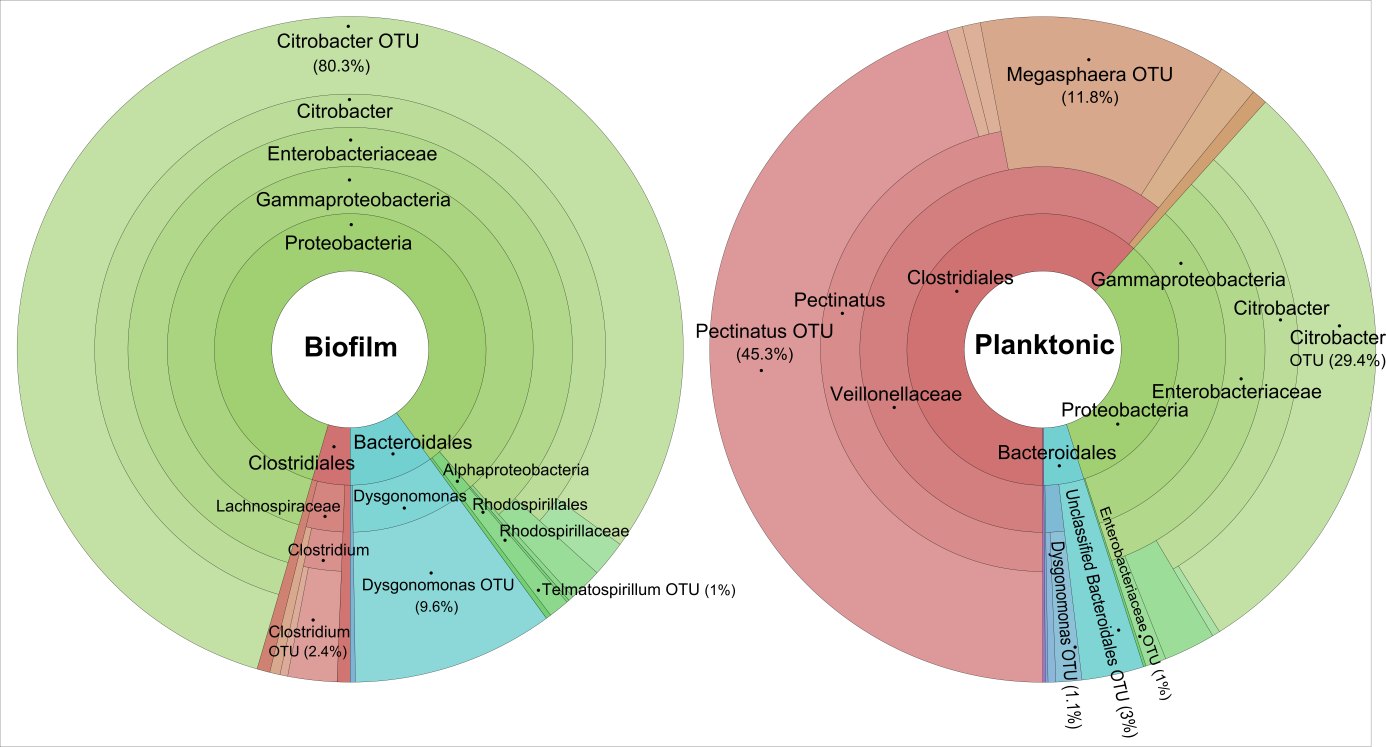


Fig. S4. Microbial composition of the biofilm and planktonic community in the cathode of BES reactor with ‑10 A·m^-2^ current at day 30. This figure only shows the abundance of populations representing more than 1% of the community in the 16S rRNA gene amplicon pyrosequencing.

**Fig. S5. FISH image for the cathodic biofilm at day 159**

Probe:


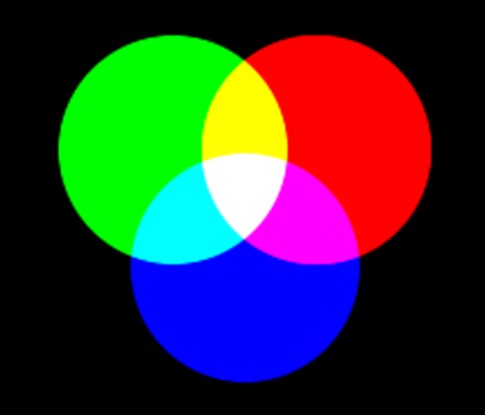
Bacteria (EUB MIX), Archaea (ARC 915), Methanosaeta (MX 825), Gammaproteobacteria (GAM MIX)

Final color:

Blue: Bacteria

Purple (blue + red): Gammaproteobacteria

Green: Archaea

Yellow: (green + red): *Methanosaeta*

| 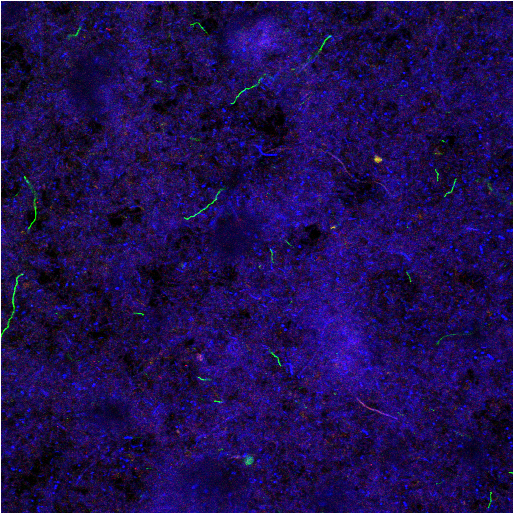 | 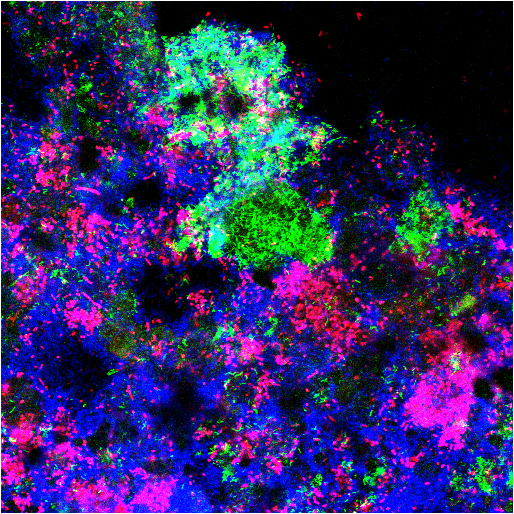 |
| --- | --- |
| Inoculum | Biofilm |

Fig. S5. FISH image of the inoculum and the cathodic biofilm at day 159. Images were analysed using DAIME Version 1.2, which indicated that 29.9% of the biofilm was colonized by Gammaproteobacteria at day 159.
